# Anaphylactic Reaction to Patent Blue Dye After Supermicrosurgical Lymphaticovenular Anastomosis for the Treatment of Secondary Lymphedema

**DOI:** 10.7759/cureus.54767

**Published:** 2024-02-23

**Authors:** Sina Heymans, Arie Sleutel, Thomas Schoeller, Laurenz Weitgasser

**Affiliations:** 1 Department of Plastic and Reconstructive Surgery, Marienhospital Stuttgart, Stuttgart, DEU

**Keywords:** lymphedema surgery, secondary lymphedema, lymphaticovenular anastomosis, patent blue dye, anaphylactic reaction

## Abstract

For decades, patent blue dye (PBV) has been utilized for sentinel lymph node biopsy and lymphatic mapping in breast cancer and melanoma staging and treatment. Fistulography and intraoperative display of lymphatic vessels for lymphaticovenular anastomosis (LVA) are frequent applications. Although its anaphylactic potential is well described, PBV is used routinely. We present the case of a 71-year-old female patient, who underwent LVA for the treatment of chronic secondary lymphedema and experienced a postoperative anaphylactic reaction including a blue-colored drug-induced maculopapular exanthema after PBV administration. This article aims to raise awareness of potential life-threatening allergic reactions and propose an alternative to PBV.

## Introduction

Lymphaticovenular anastomosis (LVA) is an established but technically challenging treatment for chronic lymphedema. Patent blue dye (PBV) allows for the easy identification of suitable lymphatic vessels and collectors, a speedier dissection, as well as the confirmation of successful anastomosis [1]. Its ease of use, low cost, and efficacy make it popular in supermicrosurgery [2]. The allergic potential of this coloring agent is well described for staging procedures in operable breast cancer [3]. To our knowledge, this is the first case report of an allergic response to PBV in lymphatic supermicrosurgery.

## Case presentation

A 71-year-old woman presented to our department with chronic secondary lymphedema of her right leg. She had undergone a melanoma resection on her right lower extremity, as well as a lymphadenectomy of her right groin, because of metastasis eight years prior. She consequently underwent radiotherapy of the right groin area. Despite conservative treatment with compression stockings and lymphatic drainage twice a week, the lymphedema was progressive and reduced the quality of our patient's life, mobility, and ability to care for herself. An indocyanine green lymphangiography confirmed a stage II lymphedema with present lymphatic collectors of the lower and upper leg. The indication for surgical treatment using LVAs was established. 

At the beginning of the operation, 1 ml PBV was infiltrated in the first and third interdigital spaces of the right foot as well as 10 cm below the right knee medially. Three end-to-end LVAs (one at the knee, two at the foot) could be carried out. The PBV flow into the venous system and the patency of the anastomosis were validated intraoperatively. The surgery finished uneventfully, and the patient was extubated.

**Figure 1:**
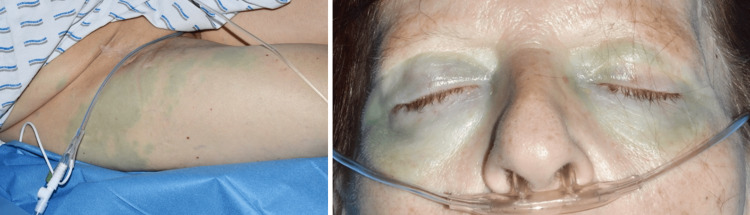
PBV-induced blue-colored maculopapular exanthema on the thigh (left) and eyelids (right) PBV: patent blue dye

In the early postoperative phase in the recovery room, about half an hour after the operation, the patient developed an anaphylactic reaction with the following symptoms: tachycardia (108/min), hypertension (169/103 mmHg), cold sweating, swelling of the eyes, shivering, as well as blue coloring of her skin and sclerae resembling a maculopapular exanthema (Figure 1) of the extremities, trunk, and eyelids. Furthermore, she developed a metabolic acidosis (pH 7.24) with a base excess of -6.6 mmol/l and a reduced bicarbonate of 18.7 mmol/l.

After the application of 250 mg prednisolone, 0.5 mg adrenaline, and an H1 and H2 receptor blocker, the patient was stabilized and transferred to our intensive care unit for surveillance. Soon after the antiallergic drug administration, her symptoms including the rash improved quickly. The further course of her stay progressed without any other complications. On the evening of the surgery day, the patient was transferred to the regular inpatient ward. Over the following days, the blue coloring of her skin and urine vanished entirely. 

In the follow-up consultations, the patient was satisfied and demonstrated lymphedema reduction of 2 cm of circumference around her lower and upper leg with improved quality of life, mobility, and overall relief of heaviness and tightness of her leg. 

## Discussion

PBV is a blue synthetic triphenylmethane dye. As a food additive, used for food coloring mainly of sweets and beverages within the European Union, it is also known as E131. In medicine, it is used in sentinel node biopsy and lymphangiography and to color lymph vessels in supermicrosurgery [4]. First allergic reactions after lymphangiography were reported in the early 1960s, and an incidence of anaphylactic reactions in about one in 1000 cases was suggested [5]. Currently, it is described that blue dyes can cause interoperative anaphylactic reactions in up to 2.7% of the patients [6]. Usually leading to mild reactions, severe and life-threatening shock is reported in 0.06% of the cases [7]. Without the pathophysiological pathways being understood, IgE-mediated reactions as well as non-IgE-mediated reactions have been suggested. Also, a sensitization to E131, being present in food, textiles, and cosmetics, and a hypersensitivity reaction after the first exposure to PBV in surgery were described [4]. Because of its possible allergic potential, PBV, as a food coloring agent, has been banned in the United States, Canada, Norway, and Australia [8,9]. In our department, PBV is used for staining lymphatic vessels during LVA surgery. Therefore, it is injected subdermally and taken up readily by draining lymphatic vessels. PBV is selectively absorbed into the lymphatics, which in our case might be deemed as indirect proof of the patency of the anastomosis as it led to a systemic anaphylactic reaction. Also, PBV is excreted into the urine and bile explaining the discoloration of the urine [6]. The visualization of the lumen of the lymphatic vessel with the blue dye makes the anastomosis possible, as it identifies the small-caliber and thin-walled lymphatic vessels. The dissection is simpler and hence shortens overall theater time [1]. Its use is effective and helpful to the surgeon, but potential allergic side effects present a certain drawback. Methylene blue dye is proposed as an alternative which is supported by the available literature. The latter is a heterocyclic aromatic chemical compound and structurally unrelated to the synthetic triphenylmethane dye PBV; therefore, there is no risk of cross-reactivity [4]. Methylene blue is widely available, is less expensive, and has a lower risk of anaphylaxis compared to PBV. Furthermore, no inferiority in displaying the lymphatic system, for instance, in sentinel lymph node biopsy, was observed [10]. 

## Conclusions

Awareness of potential life-threatening allergic reactions to PBV should be raised to lymphatic surgeons. An alternative use of methylene blue, which is structurally not related to PBV and should not lead to cross-reactivity, is therefore recommended. A direct comparison between methylene blue and PBV in the specific area of LVA has not been demonstrated yet; further studies to validate the same level of effectiveness between the two dyes are encouraged.
